# The Composites of Graphene Oxide with Metal or Semimetal Nanoparticles and Their Effect on Pathogenic Microorganisms

**DOI:** 10.3390/ma8062994

**Published:** 2015-05-27

**Authors:** Lukas Richtera, Dagmar Chudobova, Kristyna Cihalova, Monika Kremplova, Vedran Milosavljevic, Pavel Kopel, Iva Blazkova, David Hynek, Vojtech Adam, Rene Kizek

**Affiliations:** 1 Department of Chemistry and Biochemistry, Faculty of Agronomy, Mendel University in Brno, Zemedelska 1, CZ-613 00 Brno, Czech Republic; E-Mails: oliver@centrum.cz (L.R.); dagmar.chudobova@centrum.cz (D.C.); kriki.cihalova@seznam.cz (K.C.); mkremplova@volny.cz (M.K.); grizlidripac@gmail.com (V.M.); paulko@centrum.cz (P.K.); iva.blazkova@seznam.cz (I.B.); d.hynek@email.cz (D.H.); vojtech.adam@mendelu.cz (V.A.); 2 Central European Institute of Technology, Brno University of Technology, Technicka 3058/10, CZ-616 00 Brno, Czech Republic

**Keywords:** *Escherichia coli*, graphene oxide, metal or semimetal based nanoparticles, *Staphylococcus aureus*, antibacterial effect

## Abstract

The present experiment describes a synthesis process of composites based on graphene oxide, which was tested as a carrier for composites of metal- or metalloid-based nanoparticles (Cu, Zn, Mn, Ag, AgP, Se) and subsequently examined as an antimicrobial agent for some bacterial strains (*Staphylococcus aureus* (*S. aureus*), methicillin-resistant *Staphylococcus aureus* (MRSA) and *Escherichia coli* (*E. coli*). The composites were first applied at a concentration of 300 µM on all types of model organisms and their effect was observed by spectrophotometric analysis, which showed a decrease in absorbance values in comparison with the control, untreated strain. The most pronounced inhibition (87.4%) of *S. aureus* growth was observed after the application of graphene oxide composite with selenium nanoparticles compared to control. Moreover, the application of the composite with silver and silver phosphate nanoparticles showed the decrease of 68.8% and 56.8%, respectively. For all the tested composites, the observed antimicrobial effect was found in the range of 26% to 87.4%. Interestingly, the effects of the composites with selenium nanoparticles significantly differed in Gram-positive (G^+^) and Gram-negative (G^−^) bacteria. The effects of composites on bacterial cultures of *S. aureus* and MRSA, the representatives of G^+^ bacteria, increased with increasing concentrations. On the other hand, the effects of the same composites on G^−^ bacteria *E. coli* was observed only in the highest applied concentration.

## 1. Introduction

In food and textile industries or in hospital facilities, we meet technological processes that should prevent the spread of bacteria between staff, consumers and patients [1,2,3]. The necessity is the treatment of surfaces, tools and working materials by decontaminants. Application of antibiotics is not always a suitable choice, since they can be transferred into the treated material [3]. Organic compounds used for disinfection have some disadvantages including toxicity to the human body [4].

Another problem arising from using of antibiotics is a presence of bacterial strains resistant towards applied chemical substances [5,6]. The resistant bacteria are more likely to be present in hospital facilities, where genetic mutations in bacteria occur due to the long-term administration of antibiotics. This phenomenon leads to the development of various defensive mechanisms able to inactivate the applied chemical substance [7]. The most common resistant strain, which evolved from the *Staphylococcus aureus*, is methicillin-resistant *S. aureus* (MRSA) [8,9]. The incidence of MRSA in hospitalized patients results particularly from an intensive and long-term therapy [10], where *S. aureus* is exposed to relatively high concentrations of antibiotics for a longtime, thus certain mutations can develop.

Sterile surfaces, tools, fabrics or packaging are absolutely necessary in the aforementioned areas, and it is therefore essential to ensure their treatment with antibacterial agents. Excluding conventional antibiotics, such effects were described in the case of metal nanoparticles [11], graphene oxide (GO) or biofunctionalized graphene [12].

GO is similar to the graphene sheet; it is a modified form of graphene, which contains exogenous oxygen-containing functional groups on its surface. The functional groups allow the application of broad spectrum of substrates to form thin layers (films) or networks [13,14]. GO as well as metal nanoparticles exhibit wide antimicrobial activity against bacterial pathogens [15,16,17]. Graphene with antimicrobial effect is used in food packaging or for the coating of biomedical devices, where the bacterial colonization of a surface is undesirable [18]. The modifications of graphene with antibacterial metal nanoparticles render this phenomenon more effective.

The production of nanosized material as a potential antibacterial agent is thus very important, since it can be used as a suitable alternative to overcome the multidrug resistance of several organisms. The aim of this study was to develop composites, which are themselves, partially, antimicrobial agents. The packing of metal or metalloid nanoparticles to the carrier based on the GO may lead to a gradual release of nanoparticles and therefore to the long-term effects of nanoparticles on various bacterial strains as it was shown in presented study.

## 2. Results and Discussion

The objective of the present study was the preparation of synthesized graphene oxide-based composites with various types of metals- and metalloids-based nanoparticles. Individual parts of designed composites exhibit antimicrobial effects [6,7,17,19], so their use as the composite building blocks can synergistically increase the antimicrobial activity and provide promising materials to control and eliminate bacterial infections.

The state of GO-based composites after drying their dispersions can be easily examined using Scanning Electron Microscopy (SEM) micrographs. This method also enabled determining the degree of exfoliation, which is crucial for nanoparticular character. The SEM micrographs also allowed the rating of metal- or metalloids-based nanoparticles adhesion to GO-based carrier. Many recent studies have focused on microstructure investigation of GO and related materials using SEM analysis due to the facile evaluation possibility of the structure and quality of source or synthesized materials, as well as the assessment of material modifications [20,21,22,23,24]. SEM Elemental Mapping and SEM line scan analysis provide important information about metal- and metalloid-based nanoparticles distribution on the surface of GO-based carrier [25,26,27,28]. The nature of prepared composites was verified by Dynamic Light-Scattering (DLS), *i.e.*, directly in the solution, without the drying step. The comparison and evaluation of size changes of graphene oxide-based composites and sheets size distribution is preferably carried out directly in the solution.

The results obtained by SEM and DLS were confronted with micrographs captured using Ambient Light Microscopy (LM), which did not allow the study of nanoparticles, but allowed a comfortable visualization of the particle size on the micrometer level (which is a typical particle size for GO and related materials), which is a faster and cheaper alternative to the low-magnification SEM images [29]. The concentration of free metal ions in the solutions was determined electrochemically by Differential Pulse Voltammetry (DPV). The detected concentrations corresponded to non-reduced metal or metalloid, which was not present in the form of nanoparticles (or which did not precipitated completely in the form of insoluble salt in the case of GO-AgP). The total metal concentration in the prepared composite materials was verified by Atomic Absorption Spectrometry (AAS).

**Figure 1:**
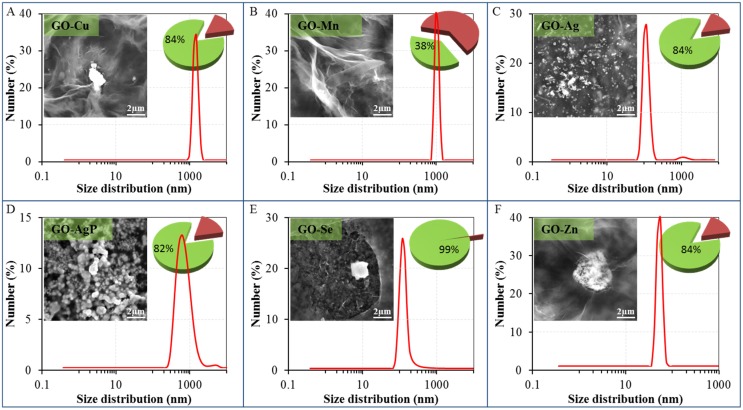
Dynamic light-scattering (DLS) spectra of graphene oxide (GO) composites with metal- or metalloid-based nanoparticles dispersions—size distribution by number: (**A**) GO-Cu; (**B**) GO-Mn; (**C**) GO-Ag; (**D**) GO-AgP; (**E**) GO-Se; and (**F**) GO-Zn. The size of composites was evaluated by Zetasizer Nano NS with a scattering angle θ = 173°. At least 200 particles per sample were measured to obtain the size distribution (the standard spherical particle models were used in DLS). Inserts show their representative SEM images by drying composites dispersions on clean silicon wafers. The length of scale bar is 2 µm. Pie charts show the amount of reduced or precipitated metal or metalloid (green sectors with values) and metal or metalloid ionic form (russet sectors) determined by DPV. The amount of reduced or precipitated metal or metalloid was determined as the difference between total applied amount and amount found by DPV.

The SEM micrographs (Figure 1, Figure S1 in supplementary) confirmed the preservation of the original structure of the large area of GO [17,30,31,32], which remained maintained in comparison with starting material and which is in agreement with already published structure of nanocomposites based on GO with Cu [33], Mn [34], Ag [35,36,37], AgP [38] and Zn [39]. In the case of Cu, Ag, Se and Zn composite, there were also observable larger particles attached on the surface with an approximate diameter of 0.5 µm (Figure 1A,D–F). This phenomenon is very similar to some already described composites, e.g., in the case of Ni nanoparticles [40]. Also smaller particles, with dimensions of tenths or units of nm, were present, which were observed for example in the case of silver nanoparticles [35] and others [28]. The particles containing Mn were not noticeable on the surface of GO and were therefore more likely to be uniformly dispersed on the carrier surface (Figure 1B; Supplementary Figure S3A). It is obvious that the particles based on Cu, Mn and Zn did not consist of zero-valent metal, but due to their reactivity, they were comprised of some oxygenated groups and these metals in nanoparticles were presented in their oxidized form. In the case of composite with elemental silver, GO formed layered, transparent structure, which cannot be visible due to a massive coverage with silver Ag(0) nanoparticles with dimensions of hundreds of nanometers (Figure 1C; Supplementary Figure S2A). Also in the case of GO-AgP, the original structure of GO was not clearly observable on SEM micrographs, since the carrier surface was fully covered with microscopic coral structure formed by insoluble silver phosphate (with dimensions of approximately one micrometer, Figure 1D; Supplementary Figure S2B, and containing Ag (I)). The original structure of GO was retained in the case of zinc-based material (Figure 1F) and agglomerates of small particles containing applied metal were attached on the surface. Only in the case of selenium-containing composite, the original structure of GO carrier was partially corrupted. The reduced selenium particles observed by SEM had a size from a few tenths of nanometers to a few micrometers (Figure 1E; Supplementary Figure S3B) and consisted of elemental selenium, *i.e.*, Se(0), according to SEM Line Scan Analysis (Supplementary Figure S4).

The SEM Elemental Mapping confirmed the uniform distribution of reduced nanoparticles on the surface of the carrier in the cases of GO-Cu, GO-Mn, GO-Ag, and Go-AgP (Supplementary Figures S2 and S3), however some larger particles were also observed. In the case of GO-AgP composite, the SEM Elemental Mapping proved that the concentrations of silver, oxygen and phosphorus copied the morphology of examined area in all three cases and these records were therefore the sufficient proof of GO-AgP composite compositions. The significant portion of coral-like particles of silver phosphate fulfilled the criterion of nanoparticles. SEM line scan analysis performed on GO-Se composite (Supplementary Figure S4) proved the existence of discrete selenium particle wrapped with a thin layer GO carrier [41].

**Figure 2:**
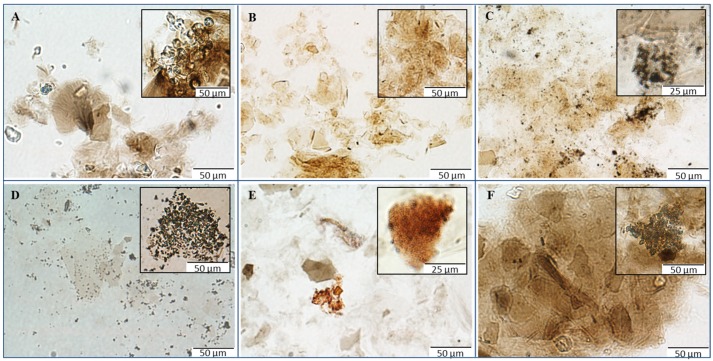
Micrographs of GO composites with metal- or metalloid-based nanoparticles using microscopy in ambient light: (**A**) composites of GO with copper-based nanoparticles; (**B**) composites of GO with manganese-based nanoparticles; (**C**) composites of GO with silver nanoparticles; (**D**) composites of GO with silver phosphate nanoparticles; (**E**) composites of GO with selenium nanoparticles; and (**F**) composites GO with zinc-based nanoparticles. The inverted system microscope Olympus IX 71 (Tokyo, Japan) with the total magnification 400× was used for the analysis. The images were captured by Olympus Camera DP73 and processed by Olympus Stream Basic 1.7 software with the images resolution 1600 × 1200 pixels and the length of scale bar 50 µm or 25 µm, respectively.

LM records obtained directly from the solutions of prepared materials represent quite a significant contribution to the understanding and characterization of the structures of synthesized materials. These micrographs allowed assessing the condition of the GO carrier and metal- or metalloid-based nanoparticles and/or their insoluble compounds (Figure 2). GO-Cu and GO-Mn composites exhibited fully retained original structure of the GO carrier (Figure 2A,B). In the case of GO-Ag composite, agglomerates of small silver particles could be observed on the coated and uncoated surface of the carrier (Figure 2C). On micrographs of GO-AgP, some uncovered sheets of GO can be seen (Figure 2D), nevertheless other sheets of GO carrier were strongly covered by silver phosphate particles (Figure 2D insertion). Thus, it can be stated that in both cases of silver-based composites, the character of GO carrier remained unchanged, in accordance with original structure. In contrast to the previous composites, GO-Se showed a significant decrease in size of larger GO particles, which is visible on micrographs (Figure 2E); the presence of the red-colored elemental selenium particles with various degree of agglomeration is also noticeable, revealing a successful reduction of selenite employed for coating. Discrepancy in original GO carrier can also be registered in the case of GO-Zn composite (Figure 2F), where huge agglomerates of GO sheets with hundreds of µm in diameter can be seen in micrographs. The aggregation is most likely to be triggered by the coordination between GO and divalent ions. Finally, it is worth mentioning that all the observations from LM are in good correlation with other carried experiments.

The DLS results complemented the findings discussed above (Figure 1). For the study of GO-based materials, DLS technique can be used, but it should be taken into account that dimensions of GO sheets and GO-based nanocomposites determined by this technique do not represent the real particle sizes [30]. The characterization of the original structure of GO was in complete agreement with results published previously by Lee and coworkers [42]. In the case of GO-Cu and GO-Mn composites, only particles with the size and distribution well corresponding to the starting material could be observed. In the context of the results from other techniques, it can be expected that metal-based particles have small diameters and are adhered to the supporting surface of GO carrier. The bimodal particle size distribution in the case of GO-Ag detected by DLS was consistent with the results obtained by SEM. The DLS results showed the presence of silver nanoparticles with a size of approximately 80–200 nm and the presence of bigger particles, which corresponded to the GO carrier. Similar type of material was reported to have a thickness even less than 1 nm or a thickness of a few nanometers only (planar GO tickets dimension) and diameter at the level of tens to hundreds and/or thousands of nanometers (400 nm–2 µm) determined by Atomic Force Microscopy [43]. Analogous results were reported in other studies, where DLS was utilized [17,32]. Since the GO-Ag solution exhibits two different particle size distributions, it is clear that the part of Ag particles was coated on carrier surface and the remaining part of the silver nanoparticles remained in the form of discrete particles. The bimodal distribution could be found in the case of GO-AgP, too, so similarly, it can be expected that a minor proportion of silver phosphate precipitate was present in the solution in a form of discrete nanoparticles. In the case of GO-Se, a significant decrease in the particle size was observed, which is connected with the disruption of integrity of the carrier. The asymmetrical shape of the signal indicated that the examined system might be more complicated and the signal pattern might be the result of the concurrent presence of a certain proportion of bulkier discrete selenium nanoparticles in solution and the remaining proportion presented the selenium coated to GO carrier. In the case of GO-Zn, it was possible to detect by DLS only nanoparticles having the size below 100 nm (Figure 1F), so it can be expected that this response was related to unbound condensed Zn-based nanoparticles. Conversely, the lack of response connected to GO carrier, or composite particles, probably reflects the condensation of GO-based particles and the formation of agglomerates at the level of the microparticles (Figure 2F). A similar behavior has already been described in the case of copper ions [43]. 

**Figure 3:**
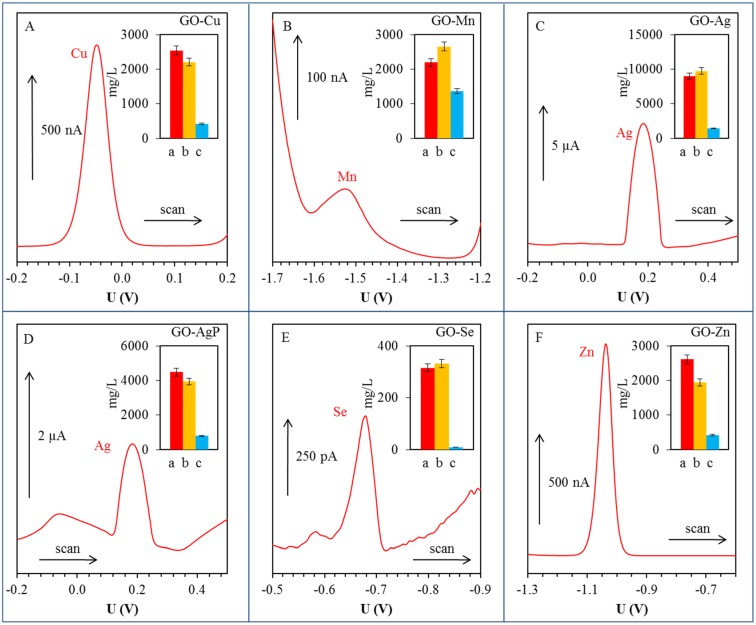
Differential pulse voltammograms of free (non-bonded) metal or metalloid ions in supernatants: (**A**) Cu(II); (**B**) Mn(II); (**C**) Ag(I); (**D**) Ag(I); (**E**) Se(IV); and (**F**) Zn(II). Detailed parameters of measurements are described in section Materials and Methods 3.5. In inserted graphs: red column (**a**) corresponds to applied concentration (mg/L); orange column (**b**) corresponds to total (free and bonded) metal concentration (mg/L) determined by atomic absorption spectrometry; blue column (**c**) expresses concentration of free ions in the solution (mg/L) by differential pulse voltammetry.

Metal ions were detected in their aqueous solutions (supernatants) by anodic or cathodic stripping differential pulse voltammetry [44,45,46,47]. Except silver ions, the metal ions were detected by using a hanging mercury drop electrode (HMDE). The electrochemical peaks of Cu(II), Mn(II), Se(IV) and Zn(II) were evaluated at the potential of −0.05, −1.68, −0.68 and −1.04 V, respectively. Silver was detected as Ag(I) by glassy carbon electrode, and the electrochemical signal of Ag(I) was observed at the potential +0.18 V. The above-mentioned methods allowed the detection of given metals in their non-reduced and unprecipitated form (Figure 3). Significant concentrations of metal ions were detected only in GO-Mn solutions. In the case of Cu, the electrochemical measurements showed that about 16% of copper compound used for synthesis was not reduced under given conditions and ionic form of metal is still present in the solution in the form of Cu(II) ions. In the case of GO-Mn, it was electrochemically detected that 62% of used Mn(II) in GO-Mn solution remained unreacted. Based on the electrochemical determination, it can be confirmed that the silver in the form of Ag(I) remained non-reduced in solution from 16% and the amount of unprecipitated silver in the case of GO-AgP corresponded to 18% of originally loaded quantity. Very low Se(IV) concentrations detected in the solution corresponded to 0.6% of the original amount and showed almost 100% efficiency of the reduction process. The quantity of non-reduced Zn(II) determined electrochemically corresponded to 16% of the initial amount.

Nowadays, researchers have turned their attention towards the biological application of GO nanosheets [48]. In recent years, nanostructured materials are highly recognized as antibacterial agents due to the multi-drug resistant bacteria [19]. Graphene-based materials also have antibacterial properties. *Hu et al.* reported the antibacterial activity of the graphene and GO towards *E. coli* [49]. It was previously shown that the merging of graphene-based carriers with metal- or metalloids-based nanoparticles multiplies the antibacterial effect of resulting composite [50]. The packaging of nanoparticles into the carrier based on GO can result in a gradual release of nanoscaled particles leading from gradual disintegration of GO, thus a longer-term effect of composite on the bacterial strains can be achieved. The graphene-based materials can kill bacteria in two ways. The GO kills bacterial cells through cell-wrapping especially, while reduced graphene oxide (rGO) kills bacterial cells mainly through cell-trapping. The antibacterial activity of GO and rGO has been attributed to membrane stress induced by sharp edges of graphene nanosheets, which may result in physical damages on cell membranes, leading to the loss of bacterial membrane integrity and the leakage of nucleic acid [17].

**Figure 4:**
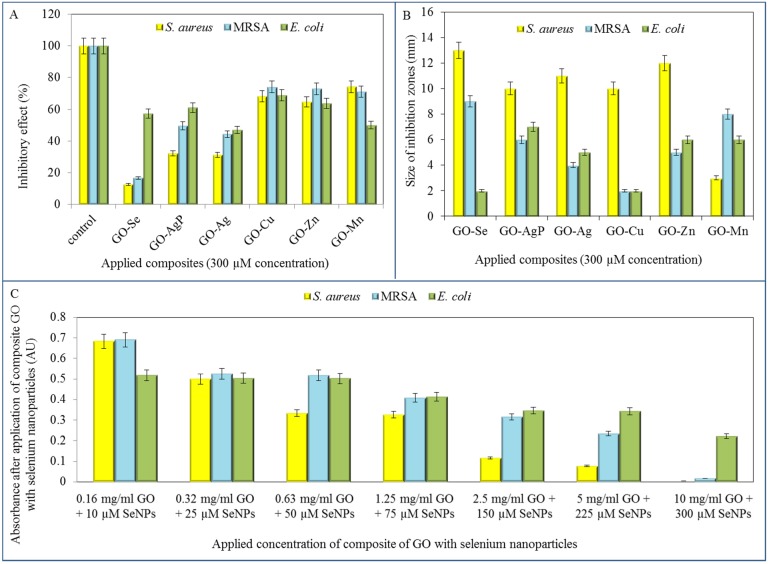
Determination of bacterial growth after the application of GO composites with metal- or metalloid-based nanoparticles. (**A**) Percentage of bacterial growth after application of 300 µM concentration of tested composite using method of growth curves after 24 h of measurement; (**B**) Determination of inhibition zones arising after application of 300 µM concentration of tested composites on *S. aureus*, MRSA and *E. coli*; (**C**) Determination of bacterial growth after the application of various concentrations of composite of GO with selenium nanoparticles.

After the detailed characterization (Table 1), the GO-based composites were tested is terms of their antimicrobial effects on *S. aureus*, MRSA and *E. coli* strains. The composites were firstly applied in the identical 300 µM concentrations on all three model organisms and the effect was subsequently evaluated spectrophotometrically by decrease in absorbance values in comparison with the control strains (no treatment). The most pronounced inhibition of *S. aureus* growth was observed after the treatment of GO composite with selenium nanoparticles, since the decrease in comparison with control was up to 87.4%. Furthermore, the highest inhibitions for the composite with silver and silver phosphate nanoparticles observed were 68.8% and 56.8%, respectively. Other tested composites did not reach such inhibitory effect, even though the reduction of *S. aureus* growth was determined (Figure 4A).

**Table 1:** Overview of some properties of synthesized GO composites.

Sample	Original Structure of GO Carrier Preserving	Nanoparticles Coating on GO Carrier	Uniformity of Particles Distribution	Metal Ions Reduction Success
GO-Cu	+++	+++	+++	++
GO-Mn	+++	+++	+++	+
GO-Ag	+++	++	++	++
GO-AgP	+++	++	++	++
GO-Se	+	+++	+++	+++
GO-Zn	+	++	+++	++

Notes: (+++) indicates a complete or nearly complete achievement of an ideal state or theoretical assumption; (++) indicates functionally insignificant deviations from the ideal state (*i.e.*, coating not less than half of the amount of nanoparticles); (+) indicates a state where the monitored indicator is not sufficiently satisfied but still does not preclude expected functionality of designed composite.

Subsequently, the method of inhibition zones was applied to examine the antimicrobial effect of the conjugates. The most significant formation of inhibition zones was determined in *S. aureus* strain treated with all of GO composites except GO-Mn. Based on the inhibition zones results, GO-Se was shown to exhibit the strongest antibacterial activity in G^+^ bacteria (13 mm in *S. aureus*, 9 mm in MRSA), while treatment of *E. coli* with GO-Se caused minimal inhibition (2 mm) (Figure 4B).

The final microbiological testing comprised the determination of growth curves of tested strains. In previous trials, the antimicrobial effects of selenium nanoparticles were already described [1,6] and their activity was confirmed also after the formation of composite with GO. The tested strains were treated with composites of the following content: concentration of GO: 0.16; 0.32; 0.63; 1.25; 2.5; 5; and 10 mg/mL and concentration of selenium nanoparticles of 0.79; 1.97; 3.95; 5.92; 11.84; 17.77; and 23.69 µg/mL (corresponds to 10; 25; 50; 75; 150; 225 a 300 µM).

In all tested bacterial strains, an increasing growth inhibition with increasing concentrations of the antimicrobial composite GO-Se was observed. The highest concentration of the composite even caused almost the total growth inhibition in resistant (MRSA) and non-resistant strain of *S. aureus*. These results were confirmed by the IC_50_ determination. The IC_50_ of GO-Se (53.5 µM of SeNPs for *S. aureus*, 93.0 µM for MRSA and 206.5 µM for *E. coli*) showed the highest concentrations for inhibition of the growth of *E. coli* bacterial culture, this strain was the most resistant to application of tested compounds. IC_50_ values were determined at 24 h of measurement. 

Noteworthy, the effect of composites with selenium nanoparticles on bacterial strains is tightly connected with their different morphology. While the effect of composites on bacterial strain *S. aureus* and MRSA, representatives of G^+^ bacteria, has been appreciable with increasing concentration, the effect of identical composites on G^−^ bacteria (*E. coli*) was negligible, except for only the highest concentration of composite (Figure 4C). This phenomenon results from composition of bacterial cell wall. While G^−^ bacteria are significantly protected with a cytoplasmic membrane and outer cell membrane, containing a thin layer of peptidoglycan between them with periplasmic compartment, all G^+^ bacteria are bound by only a single unit lipid membrane and thick layer of peptidoglycan [51]. G^+^ bacteria are thus more vulnerable to oxidative stress caused by composites containing metals or metalloids.

In terms of antimicrobial effect of composites, we are tending to the mechanism of oxidative stress on bacteria after the application of test substances, and the oxidative stress has been shown in earlier studies of silver as an antimicrobial agent [52].

## 3. Experimental Section

### 3.1. Chemicals

Chemicals used in this study (graphite, HCl, H_2_SO_4_, metal or semimetal solutions, tryptone, yeast extract, NaCl) were purchased from Sigma-Aldrich (St. Louis, MO, USA) in ACS purity unless noted otherwise. Deionized water was prepared using reverse osmosis equipment Aqual 25 (Brno, Czech Republic). Deionized water was further purified by using a MiliQ Direct QUV apparatus equipped with an UV lamp. The resistance was 18 MΩ. The pH was measured using pH meter WTW inoLab (Weilheim, Germany).

### 3.2. Preparation and Synthesis of Graphene Oxide with Nanoparticles Composite

The GO was prepared by chemical oxidation of 5.0 g graphite flakes (Sigma-Aldrich, and 100 mesh, ≥75% min) in a mixture of concentrated H_2_SO_4_ (670 mL, Sigma-Aldrich, ACS reagent 95.0%–98.0%) and 30.0 g KMnO_4_ (Sigma-Aldrich > 99%) according to the simplified Hummer’s method [53]. The reaction mixture was stirred vigorously. The oxidation process manifests outwardly by the gradual color change from dark purplish green to dark brown. After 4 days, the oxidation of graphite was terminated by slow addition of H_2_O_2_ solution (250 mL, 30 wt% in H_2_O, Sigma-Aldrich, ACS reagent) and the color of the mixture turned to bright yellow, indicating high oxidation level of graphite. Formed graphite oxide was washed 3 times with 1 M HCl (37 wt% in H_2_O, Sigma-Aldrich, ACS reagent) and repeatedly several times washed with Milli-Q water (total volume used 12 L) until constant pH value (4–5) was achieved using a simple decantation until it was possible and using centrifugation in the last steps. During the washing process with deionized water, exfoliation of graphite oxide led to the thickening of solution and formation of stable colloid of GO.

GO-Ag: A solution of silver nitrate (5 mL, 0.425 g AgNO_3_, 2.502 mM, Sigma-Aldrich > 99%) was added dropwise to the GO solution (20 mL, 7 mg/mL) under vigorous stirring. After that, Milli-Q water (5 mL) and sodium borohydride (20 mg NaBH_4_, 0.529 mM, Sigma-Aldrich, ACS) was added slowly to the reaction mixture and the resulting mixture was stirred intensively 24 h at room temperature to allow reduction.

GO-AgP: The solution of silver nitrate (2.5 mL, 0.213 g AgNO_3_, 1.251 mM, Sigma-Aldrich > 99%) and the solution of disodium hydrogen phosphate (2.5 mL, 0.335 g Na_2_HPO_4_·7H_2_O, 1.250 mM, Sigma-Aldrich, ACS reagent, 98.0%–102.0%) were poured simultaneously into the GO solution (20 mL, 7 mg/mL) under vigorous stirring. The resulting reaction mixture was stirred intensively 24 h at room temperature to allow precipitation.

GO-Se: A solution of sodium selenite (5 mL, 0.0263 g Na_2_SeO_3_·5H_2_O, 0.100 mM, Fluka > 95%) was added dropwise to the GO solution (20 mL, 7 mg/mL) under vigorous stirring. After that, sodium borohydride (20 mg NaBH_4_, 0.529 mM, Sigma-Aldrich, ACS) was added slowly to the reaction mixture under vigorous stirring and the resulting mixture was stirred intensively 24 h at room temperature to allow reduction.

GO-Mn: A solution of manganese acetate (5 mL, 0.245 g Mn(OAc)_2_·4H_2_O, 1.000 mM, Sigma-Aldrich > 99%) was added dropwise to the GO solution (20 mL, 7 mg/mL) under vigorous stirring. After that, sodium borohydride (20 mg NaBH_4_, 0.529 mM, Sigma-Aldrich, ACS) was added slowly to the reaction mixture under vigorous stirring and the resulting mixture was stirred intensively 24 h at room temperature to allow reduction.

GO-Cu: The synthesis of GO-Cu was performed according to a procedure used for GO-Mn preparation (as reported above) where only Mn(OAc)_2_·4H_2_O was replaced by copper acetate monohydrate (5 mL, 0.1997 g Cu(OAc)_2_·H_2_O, 1.000 mM, Sigma-Aldrich > 98%).

GO-Zn: The synthesis of GO-Zn was performed according to a procedure used for GO-Mn preparation (as reported above) where only Mn(OAc)_2_·4H_2_O was replaced by zinc acetate dihydrate (5 mL, 0.2195 g Zn(OAc)_2_·2H_2_O, 1.000 mM, Sigma-Aldrich > 99%).

### 3.3. Cultivation of Bacterial Strains

The bacterial strains (*Staphylococcus aureus* NCTC 8511, *Escherichia coli* NCTC 13216, methicillin-resistant *Staphylococcus aureus* ST239:SCCmec IIIA) were obtained from the Czech Collection of Microorganisms, Faculty of Science, Masaryk University, Brno, Czech Republic. The strains were stored as a spore suspension in 20% (*v*/*v*) glycerol at −20 °C. Prior to use in this study, the strains were thawed and the glycerol was removed by washing with distilled water. The composition of cultivation medium was as follows: tryptone 10 g/L, yeast extract 5 g/L, NaCl 5 g/L, sterilized MilliQ water with 18 MΩ. pH of the cultivation medium was adjusted at 7.4 before sterilization. The sterilization of the media was carried out at 121 °C for 30 min. using a sterilizer (Tuttnauer 2450EL, Beit Shemesh, Israel). The prepared cultivation media were inoculated with bacterial culture into 25 mL Erlenmeyer flasks. After the inoculation, the bacterial cultures were cultivated for 24 h on a shaker at 600 rpm and 37 °C. The bacterial culture, cultivated under these conditions, was diluted by cultivation medium to OD600 = 0.1 and used in the following experiments. 

### 3.4. Characterization of Composites

#### 3.4.1. Scanning Electron Microscopy

The structures of the composites of rGO with silver, silver phosphate, zinc, selenium, copper and manganese nanoparticles were characterized by SEM. For documentation of the nanoparticles structure, a MIRA3 LMU (Tescan, Brno, Czech Republic) was used. This model is equipped with a high brightness Schottky field emitter for low noise imaging at fast scanning rates. The SEM was fitted with In-Beam SE detector. For automated acquisition of selected areas a TESCAN proprietary software tool called Image Snapper (Tescan, Brno, Czech Republic) was used. The software enabled automatic acquisition of selected areas with defined resolution. An accelerating voltage of 15 kV gave satisfactory results regarding maximum throughput.

#### 3.4.2. Characterization of Particle Size

The average particle size and size distribution were determined by quasielastic laser light scattering with a Malvern Zetasizer (NANO-ZS, Malvern Instruments Ltd., Worcestershire, UK). A 1.5 mL distilled water solution of the nanoparticle (1 mg/mL) was put into a polystyrene latex cell and measured at detector angle of 173°, wavelength of 633 nm, refractive index of 0.30, real refractive index of 1.59, and temperature of 25 °C.

#### 3.4.3. The Microscopy of Composites in Ambient Light

The inverted system microscope Olympus IX 71 (Olympus, Tokyo, Japan) with the objective LucPlanFLN Mag. 40× (N.A. 0.6; F.N. 22; W.D. 3-4.2 was used for the imaging of composites. The images were captured by Camera Olympus DP73 and processed by Stream Basic 1.7 Software (Olympus, Tokyo, Japan), the images resolution was 1600 × 1200 pixels.

### 3.5. Differential Pulse Voltammetry

The determination of zinc, copper, selenium and manganese by differential pulse voltammetry were performed with 797 VA Computrace (Metrohm, Switzerland), using a standard cell with three electrodes. A hanging mercury drop electrode (HMDE) with a drop area of 0.4 mm^2^ was the working electrode. An Ag/AgCl/3M KCl electrode was used as reference and platinum electrode as auxiliary. The analyzed samples were deoxygenated prior to the measurements by purging with argon (99.999%). For zinc, copper and manganese detection, 0.2 M acetate buffer (pH 5) was used as a supporting electrolyte. The parameters for zinc and copper determination were as follows: initial potential −1.3 V, end potential 0.2 V, purge time 90 s, deposition potential –1.15 V, deposition time 240 s, pulse amplitude 25 mV, pulse time 0.04 s, voltage step 5 mV, voltage step time 0.3 s, sweep rate 0.0168 V/s. For manganese detection, the initial potential was −1.7 V, end potential −1.2 V, purge time 120 s, deposition potential −1.7 V, deposition time 210 s, pulse amplitude 75 mV, voltage step 2 mV and sweep rate 0.0061 V/s. For selenium determination, 0.128 M ammonium sulfate, 0.123 mM copper sulfate and sulfuric acid (to adjust pH to 2.2) were used as a supporting electrolyte. The parameters of the measurement were as follows: initial potential of −0.4 V, end potential −0.9 V, deoxygenating with argon 120 s, accumulation time 200 s, deposition potential −0.6 V, time interval 0.05 s, voltage step 6 mV, pulse amplitude 30 mV. For the determination of all these metals, the volume of injected sample was 20 µL, and the volume of the measurement cell was 2 mL (20 µL of sample + 1980 μL electrolyte, except manganese determination: 20 µL of sample + 40 µL of Zn(II) ions (1g/L) + 1940 µL of electrolyte). 

The electrochemical determination of silver by differential pulse voltammetry was performed using a CH Instruments Electrochemical Workstation (CH Instrument Inc., Austin, TX, USA), using glass measuring cell with three electrodes. The glassy carbon electrode was the working electrode, an Ag/AgCl/3M KCl was the reference and a platinum wire was the auxiliary one. The parameters of this method were as follows: initial potential −0.2 V, end potential 0.5 V, modulation amplitude 0.05 V, step potential 1 mV. 0.2 M acetate buffer (pH 5) was used as a supporting electrolyte. The volume of injected sample was 20 µL; the volume of electrolyte was 1980 µL. For results evaluation, the software CHI 440A (CH Instrument Inc., Austin, TX, USA) was used.

### 3.6. Measurement of Inhibition Zones

To determine the antimicrobial effect of different composites based on rGO with different types of nanoparticles (AgNPs, SPNPs, CuNPs, ZnNPs, MnNPs and SeNPs) on bacterial cultures of *S. aureus*, *E. coli* and methicillin-resistant *S. aureus*, the measurement of the inhibition zones was performed. Agar surface on Petri dish was covered with a mixture of 100 mL of 24 h bacterial cultures in the exponential phase of growth, and 3 mL of LB medium (Luria Bertani medium). Excess volume of the mixture on the Petri dishes was aspirated. Discs (ϕ 1 cm) were mixed with tested solutions of composites in Eppendorf tubes. Soaked squares were then laid crosswise on a Petri dish with two discs per dish. The Petri dishes were insulated against possible external contamination and placed in a thermostat (Tuttnauer 2450EL, Beit Shemesh, Israel) set at 37 °C for 24 h. After 24 h of incubation, the inhibition zones were measured and photographed in each Petri dish [38].

### 3.7. Determination of Growth Properties

The second procedure for the evaluation of an antimicrobial effect of best composite from the determination of growth properties (rGO with selenium nanoparticles) was based on the measurement of an absorbance of *S. aureus*, *E. coli* and MRSA bacterial cultures. An apparatus Multiskan EX (Thermo Fisher Scientific, Bremen, Germany) via Ascent Software for Multiskan was used with subsequent analysis in the form of growth curves. Bacterial cultures from previous determination of growth properties were diluted with LB medium to an absorbance of 0.1 a.u. measured using a Specord spectrophotometer 210 (Analytik, Jena, Germany) at a wavelength of 600 nm. The diluted cultures were pipetted into a microplate (total volume of 300 µL) alone as a control variant, or with various concentrations of tested composite of rGO with selenium nanoparticles. The concentrations of selenium nanoparticles in this composite in the well were 0; 10; 25; 50; 75; 150; 225, and 300 μM. The measurements were carried out at time 0, then each half-hour for 24 h at 37 °C, at a wavelength of 600 nm. The measured absorbances were analyzed in a graphic form as growth curves for each experimental group individually [38].

## 4. Conclusions

The present experiments were performed to study the effects of composites based on GO with metal- or semimetal-based nanoparticles on the most common pathogenic microorganisms causing serious bacterial infections (*S. aureus*, MRSA, *E. coli*). Our results pointed to an antimicrobial effect of all tested composites on three tested pathogenic bacterial strains; however, the best results were achieved after the application of GO-Se-based composite. The microbiological methods confirmed the increasing antimicrobial activity with the increasing concentration of composites. From the results of our previous studies, and studies of other scientific groups, it can be confirmed that individual parts of the composites (GO and nanoparticles) can show antimicrobial effect. The obtained results can be useful in practice to prevent the risk of the spread and proliferation of bacterial infections. The coating of nanoparticles on GO may also lead to a slower release of nanoparticles in connection with the gradual decomposition of GO composite, including prolonged exposure of the composites at the infection site.

## Supplementary Materials

Supplementary materials can be accessed at: http://www.mdpi.com/1996-1944/8/6/2994/s1.
