# Supplementary material for: The Composites of Graphene Oxide with Metal or Semimetal Nanoparticles and Their Effect on Pathogenic Microorganisms
Source: Materials (Basel). 2015 May 27;8(6):2994–3011. doi: 10.3390/ma8062994 (PMC5455720; doi:10.3390/ma8062994)
Supplement: Supplementary file 1 [file materials-08-02994-s001.pdf]

Supplementary Materials

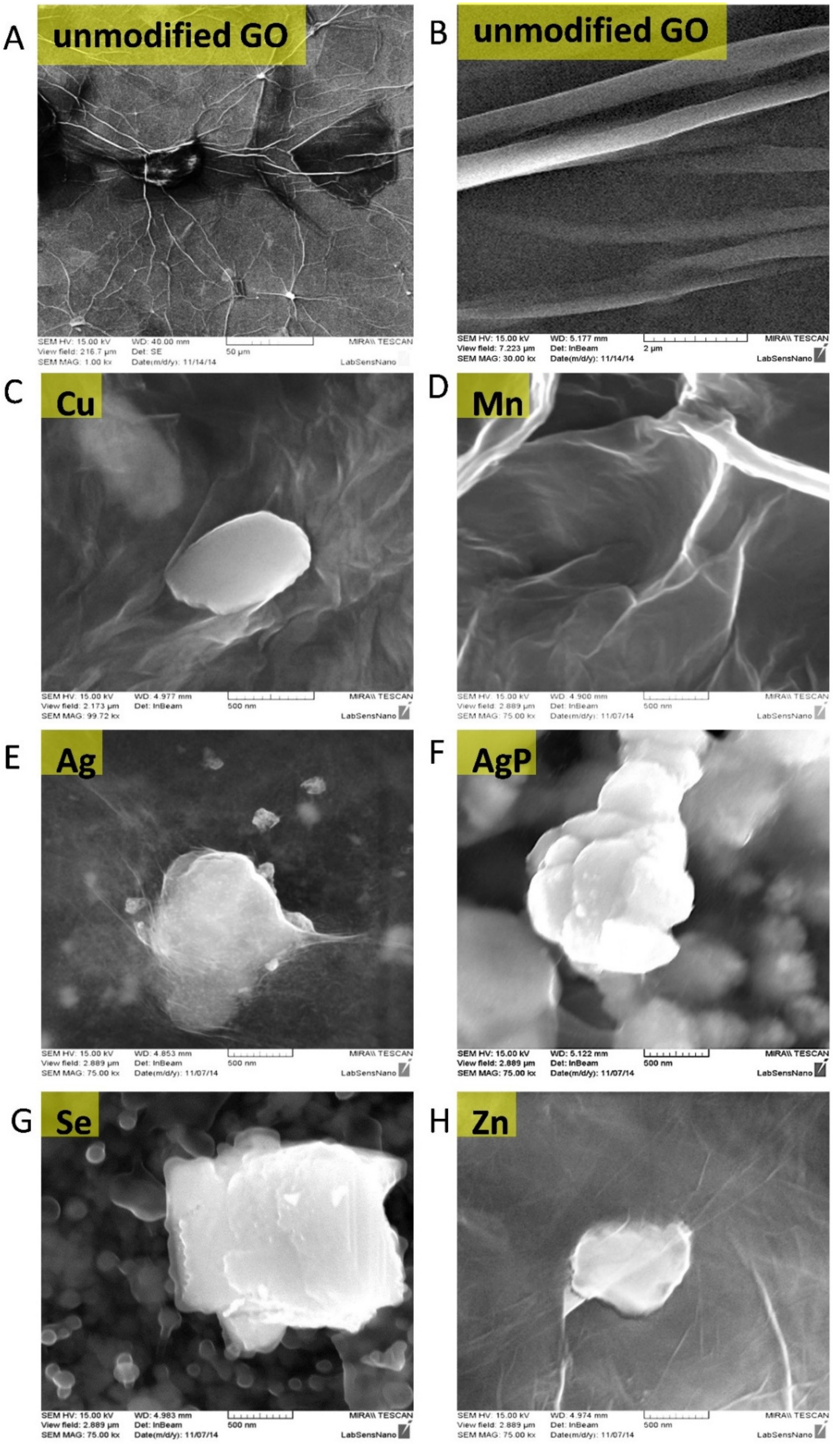

Figure S1. SEM images of GO and GO composites with metal- or metalloid-based nanoparticles obtained from dried dispersions on clean silicon wafers: (**A**) unmodified GO (magnification 1000×); (**B**) unmodified GO (magnification 30000×); (**C**) composite of GO with copper-based nanoparticles (magnification 100000×); (**D**) composite of GO with manganese-based nanoparticles (magnification 75000×); (**E**) composite of GO with silver nanoparticles (magnification 75000×); (**F**) composite of GO with silver phosphate nanoparticles (magnification 75000×); (**G**) composite of GO with selenium nanoparticles (magnification 75000×); (**H**) composite of GO with zinc-based nanoparticles (magnification 75000×).

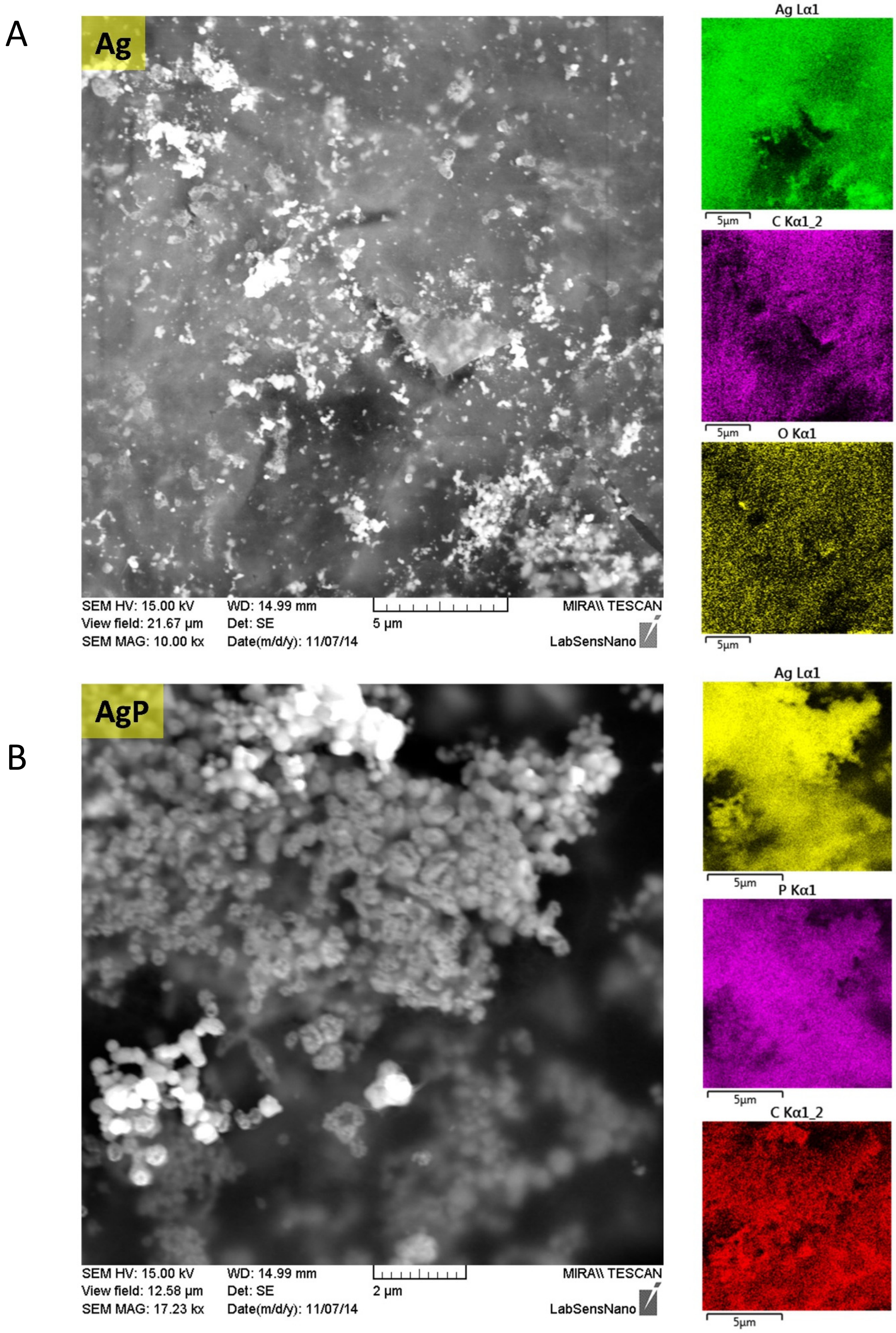

Figure S2. SEM micrographs of GO composites and SEM elemental mapping analyses: (**A**) composite of GO with silver nanoparticles (magnification 10000×) and SEM elemental mapping for Ag (green), C (violet) and O (red); (**B**) composite of GO with silver phosphate nanoparticles (magnification 17000×) and SEM elemental mapping for Ag (yellow), P (violet) and C (red).

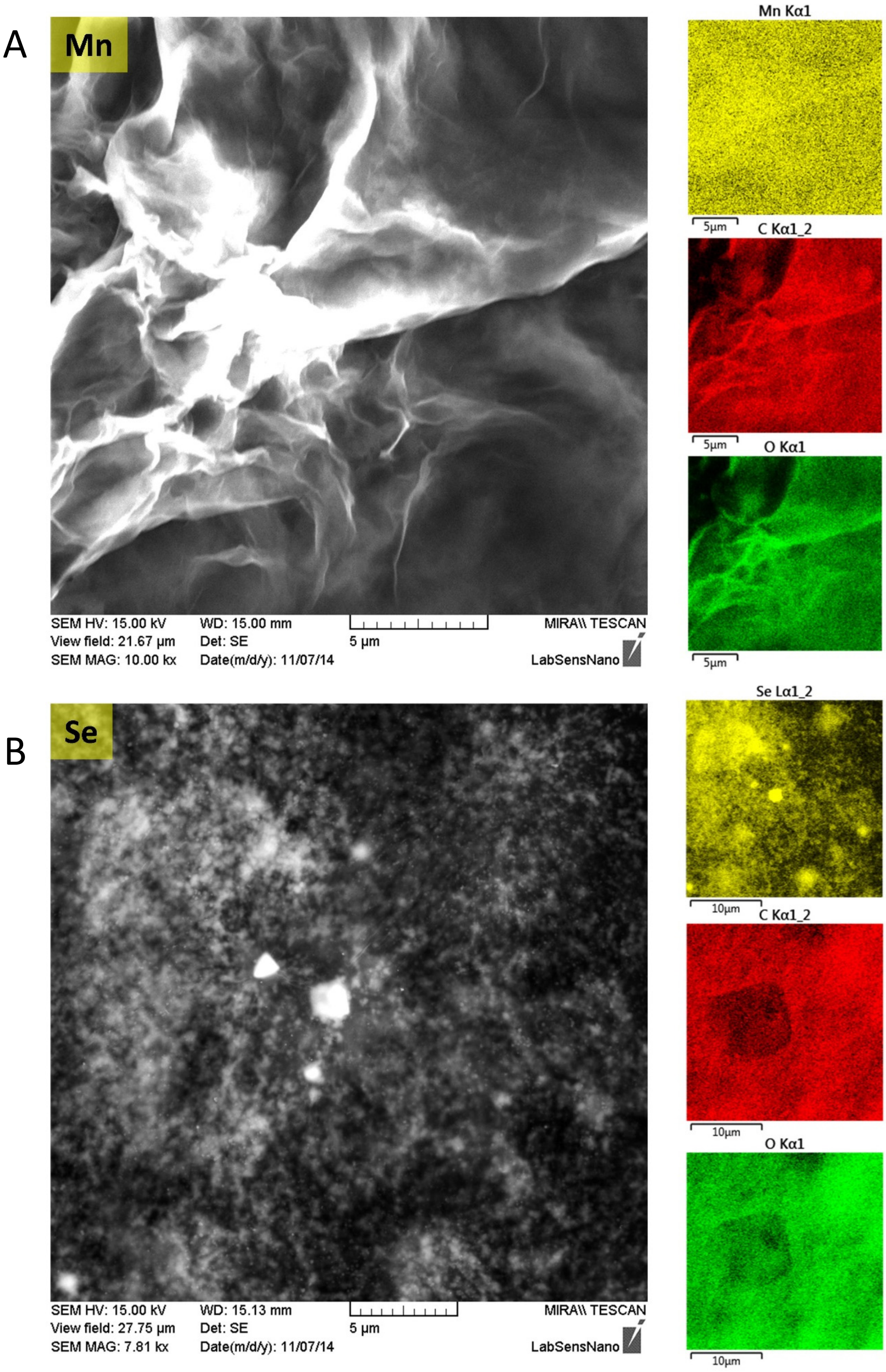

Figure S3. SEM micrographs of GO composites and SEM elemental mapping analyses: (**A**) composite of GO with manganese-based nanoparticles (magnification 10000×) and SEM elemental mapping for Mn (yellow), C (red) and O (green); (**B**) composite of GO with selenium nanoparticles (magnification 7800×) and SEM elemental mapping for Se (yellow), C (red) and O (green).

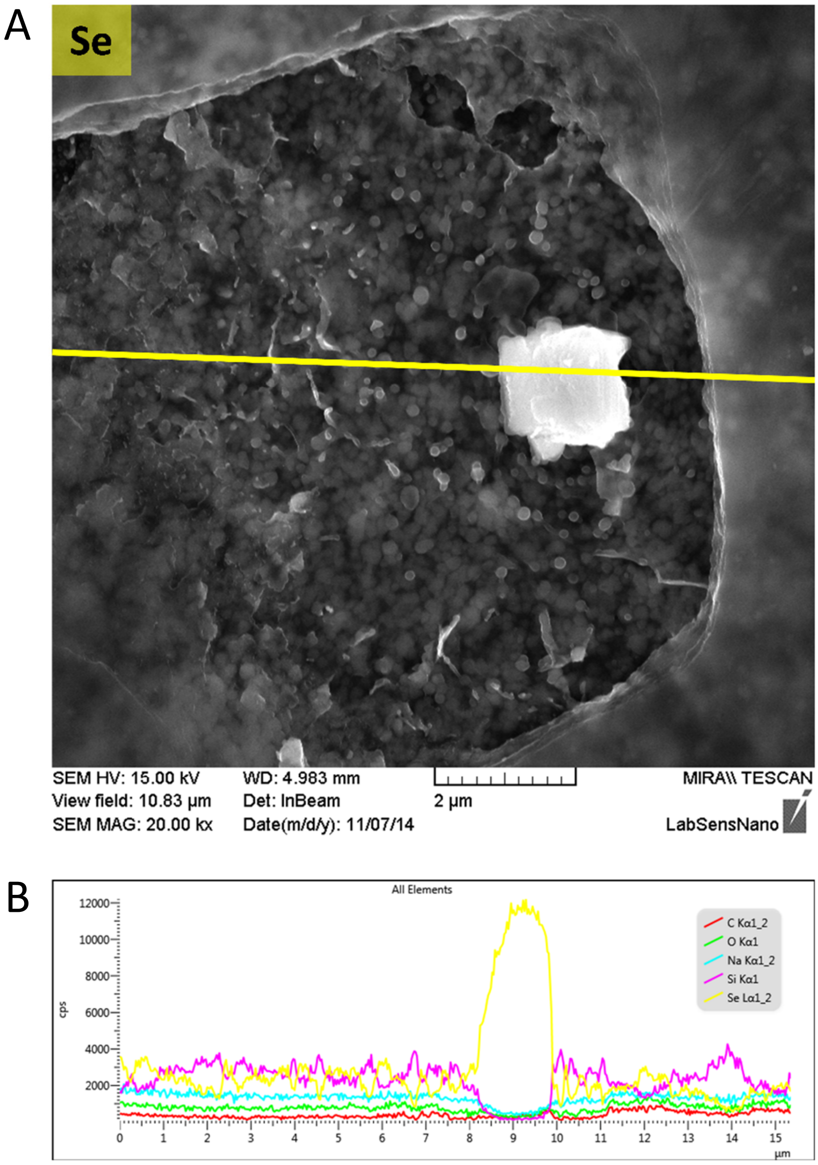

Figure S4. (**A**) EDS elemental line scan of Se particle performed on GO-Se composite; (**B**) EDS elemental concentration profiles along the yellow scan line in (**A**).
